# The association of pre-pregnancy BMI on leptin, ghrelin, adiponectin and insulin-like growth factor-1 in breast milk: a case–control study

**DOI:** 10.1017/S0007114521002932

**Published:** 2022-06-14

**Authors:** Tugce Tekin Guler, Nevra Koc, Aysun Kara Uzun, Mehmet Fisunoglu

**Affiliations:** 1 Department of Nutrition and Dietetics, Faculty of Health Sciences, Hacettepe University, Ankara, Turkey; 2 Department of Nutrition and Dietetics, Ankara City Hospital, Ankara, Turkey; 3 Department of Nutrition and Dietetics, Ankara Child Health Diseases Hematology Oncology Training and Research Hospital, Ankara, Turkey; 4 Department of Social Pediatrics, Ankara City Hospital, Ankara, Turkey; 5 Department of Social Pediatrics, Department of Nutrition and Dietetics, Ankara Child Health Diseases Hematology Oncology Training and Research Hospital, Ankara, Turkey

**Keywords:** Breast milk, Pre-pregnancy BMI, Appetite hormones, Pre-feed breast milk, Post-feed breast milk

## Abstract

The nutrient composition of breast milk alters during lactation, and maternal BMI adds more intricacy into its complexity. We aimed to compare leptin, ghrelin, adiponectin and insulin-like growth factor-1 (IGF-1) levels of pre-feed and post-feed breast milk in mothers with obesity and normal weight, and tried to determine their effects on infants’ growth over weight for length *z*-score. Twenty obese and twenty normal weight mothers with 2-month-old infants were enrolled in this case–control study. Five millilitre pre-feed breast milk and 5 ml post-feed breast milk were collected. Breast milk leptin, ghrelin, adiponectin and IGF-1 were measured by commercial kits. The pre-feed breast milk of mothers with obesity had significantly higher levels of ghrelin than mothers with normal weight (*P* = 0·025), whereas the post-feed breast milk of mothers with normal weight had higher levels of adiponectin than the mothers with obesity (*P* = 0·010). No significant differences were observed in leptin and IGF-1 levels between the two groups. Post-feed breast milk IGF-1 levels of mothers with obesity were correlated with infant’s weight for length *z*-score at 2 months (*r* −0·476; *P* = 0·034). In linear regression models, parity affected the ghrelin in pre-feed breast milk (*P* = 0·025). Our results revealed that maternal pre-pregnancy BMI was associated with breast milk components.

Childhood obesity is considered to be one of the most important public health problems of the twenty-first century. While the number of children and adolescents affected by obesity was 11 million in 1975, it became 124 million in 2016^(1)^. The number of overweight children under 5, which was 33·3 million at the beginning of the new millennium, has reached 38·9 million in 2020^(2)^. Previous studies investigating the reasons for childhood obesity found pre-pregnancy BMI and excessive weight gain during pregnancy as significant factors for increasing obesity frequencies in newborns^(3–5)^. Maternal obesity affects both the mother and the newborn, as well as the newborn’s health status in adulthood^(6,7)^. Having an obese BMI before pregnancy increases the risks of obesity, CVD, diabetes mellitus and cancer in the adult life of newborns^(8)^. Additionally, it has been argued that maternal obesity might affect breast milk composition^(9)^. Since breast milk is essential for infant nutrition, weight gain in infancy depends on breast milk intake^(10)^. The composition of breast milk is important, as rapid weight gain during infancy is a significant predictor of adiposity in later life^(11)^.

Breast milk is the optimal food source for newborns since it contains not only all micro and macro nutrients but also non-nutritional components such as hormones, antibodies and bioactive molecules. Moreover, breast milk has a dynamic composition that supports the nutritional requirements, appropriate growth, immune protection and physiological development of newborns^(12)^. On the other hand, its unique composition is extremely complex and hard to evaluate. Diurnal varieties, breast-feeding stage and many other factors contribute to this complexity. For instance, pre-feed breast milk is quite different from post-feed breast milk, whereas colostrum, transition and mature milk were disparate^(13)^.

Appetite regulating hormone levels help create a satiety signal, which terminates breast-feeding^(14)^. The primary appetite regulating hormones in breast milk, as demonstrated by previous research, are adiponectin, leptin, ghrelin, peptide tyrosine-tyrosine and glucagon-like peptide-1^(14–17)^. Moreover, it has been determined that insulin-like growth factor-1 (IGF-1), an anabolic hormone that is structurally and metabolically similar to insulin, is also found in breast milk. Its increased levels in breast milk were associated with rapid growth and obesity at later ages^(16)^. The sum of these hormones may play an important role in the regulation of acute and long-term appetite and growth of infants^(12,17)^.

The existing studies have some consensus regarding the effects of maternal BMI on breast milk leptin levels^(18)^. However, there is no consensus on how maternal BMI affects breast milk ghrelin, adiponectin and IGF-1 levels. Moreover, according to our literature review, there is also no previous study that investigates the effect of maternal BMI on the above-mentioned hormones altogether. Thus, this study aims to compare leptin, ghrelin, adiponectin and IGF-1 levels of pre-feed and post-feed breast milk in mothers with obesity and normal weight. Moreover, we also endeavoured to determine whether there is a relationship between leptin, ghrelin, adiponectin and IGF-1 levels in breast milk and early infant growth.

## Methods

### Study population and research plan

This case–control study was conducted with forty volunteer mothers who had 60 d old ((sd 10) d) newborns and fed only by breast milk since the delivery. Half of the mothers were normal pre-pregnancy BMI (18·50–24·99 kg/m^2^), and the other half were obese (BMI > 30 kg/m^2^) according to WHO BMI classification^(19)^. Participants were enrolled during their visit to Child Health and Diseases, Hematology, Oncology Training and Research Hospital for the second-month follow-up of their babies. Inclusion criteria of this study are having a healthy baby, giving vaginal delivery, delivering between 37th and 42nd weeks of pregnancy and delivering over a 2500-g baby. Any maternal health problem that might affect the breast-feeding or study results, smoking habit or alcohol consumption, pregnancy with multiples, delivering under a 2500-g baby and having preeclampsia or gestational diabetes history during pregnancy were determined as exclusion criteria. Before participating in the study, all volunteers were informed about the study and signed a written consent form. The study was conducted according to the guidelines laid down in the Declaration of Helsinki, and all procedures were approved by the Hacettepe University Non-Interventional Clinical Research Ethical Board (GO 17/843-13).

A brief study questionnaire that included socio-demographic characteristics and anthropometric measurements was applied to mothers through face-to-face interviews. After cleaning the areola, 5 ml pre-feed breast milk was collected and the mothers were asked to keep breast-feeding their infants for about 8–10 min, provided by the mother’s perceived milk availability and the infant’s feeding rate. Following the breast-feeding, 5 ml post-feed breast milk was collected from the same breast^(20)^. Breast milk was collected between 09.00 and 11.00 hours from the breast that was not used during the last breast-feeding using a Medela Swing Maxi electric breast pump into plastic bottles. All bottles were cleaned in warm, soapy water and sterilised after each usage. Collected breast milk was centrifuged and stored at –80°C after the fat layer was removed.

### Anthropometric measurements

Anthropometric measurements were taken on the same day. Pre-pregnancy body weight, weight gain during pregnancy, body weight at and after delivery were recorded from patient history forms. Maternal height was measured during the interview. Infant body weight, height and head circumference at birth were taken from the hospital records, and second-month assessments were measured during the follow-up using an infant scale, infantometer and a tape measure, respectively.

### Breast milk appetite hormone analysis

For the hormone analysis, breast milk was centrifuged, and milk fat was separated. Hormone concentrations were measured by commercially available Elisa kits (Human Elisa Kit Cloud-Clone Corp. Company; adiponectin (SEA605Hu), ghrelin (CEA991Hu), IGF-1(SEA050Hu) and leptin (SEA084Hu) in the aqueous fractions of breast milk). The kits were functioning to eliminate all other factors except the specific hormone that binds uniquely and use a reaction to generate a colour signal that can be properly quantified at a specific wavelength. The minimum detection limits for leptin, ghrelin, adiponectin and IGF-1 were 0·054 ng/ml, 29·5 pg/ml, 0·069 ng/ml and 0·067 ng/ml, respectively. The intra-assay CV was < 10 % and inter-assay CV was < 12 % for all kits. Samples were analysed in duplicate in accordance with the manufacturer’s instructions. Briefly, 100 μl of the sample was incubated with several reagents alongside manufacturer-provided standards. Hormone concentrations were measured using a Chromate 4300 plate reader (Awareness Technology Inc) and its software.

### Statistical analysis

We calculated that a sample size of 19 for each group provided an 80 % power to detect a correlation between breast milk leptin level and pre-pregnancy BMI with an *r*-value of 0·25 (5 % significance, two-sided test). On the other hand, the available data were insufficient to compute a power calculation for ghrelin, adiponectin and IGF-1. In case of any data loss during analysis, we recruited twenty volunteers for each group.

Statistical Package for the Social Sciences 22.0 package programme was used for statistical evaluation of data obtained from the study. Descriptive statistical variables are presented as mean ( 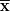 ), standard deviation, and minimum and maximum values. Mann–Whitney *U* test was used to compare the averages of data of two independent groups. Correlation analyses were performed with the Spearman correlation test and to counteract multiple testing problems, Bonferroni correction was applied. Factors that may be associated with measured hormone levels (maternal BMI, age, parity, gestational weight gain and sex) were evaluated by linear regression analysis. The results were considered statistically significant when *P* < 0·05 in 95 % CI.

## Results

This study was carried out with a total of forty lactating mothers whose ages were between 19–35 years. Characteristics of infants and mothers are given in Table 1. In our sample, the parity of mothers with obesity was significantly higher than the parity of mothers with normal weight (*P* = 0·009). Although pre-pregnancy BMI was significantly higher in mothers with obesity (*P* = 0·000), they gained less weight during pregnancy than mothers with normal weight (*P* = 0·020). Both at birth and second month weight, length and head circumference of infants were similar between groups (*P* > 0·05). Additionally, the weight for length *z*-score at 2 months was similar between groups (*P* > 0·05).

**Table 1.** Characteristics of infants and mothers (Mean values and standard deviations; median values)

	Normal weight (*n* 20) (Min–Max)			Obese (*n* 20) (Min–Max)			*P*
	Mean	sd	Median	Mean	sd	Median	
Mothers							
Age (year)	27·1	4·5	19·0–34·0	29·5	5·3	20·0–35·0	0·125
Parity	1·0		1·0–3·0	2·5		1·0–3·0	0·009**
Pre-pregnancy BMI (kg/m^2^)	22·7	2·0	18·7–24·9	31·8	2·7	30·1–40·2	0·000***
Weight at 2 months postpartum (kg)	66·6	9·0	46·0–80·0	84·7	8·6	72·0–100·0	0·000***
Length (cm)	161·7	6·6	150·0–172·0	159·5	8·3	148·0–172·0	0·357
BMI at 2 months postpartum (kg/m^2^)	25·4	2·9	19·2–32·9	33·4	3·3	25·9–42·7	0·000***
Gestational weight gain (kg)	16·2	5·6	5·0–28·0	11·3	6·3	–6·0–20·0	0·020*
Infants							
Gestational age (weeks)	39·0		37·0–42·0	38·0		37·0–40·0	0·073
Birth							
Weight (g)	3310·8	354·3	2640·0–4140·0	3323·0	337·4	2800·0–3860·0	0·850
Length (cm)	49·9	1·5	46·0–52·0	50·7	1·6	48·0–54·0	0·215
Head circumference (cm)	34·9	1·4	32·0–37·0	34·8	1·6	32·0–37·5	0·764
Second month							
Weight (g)	5449·0	787·4	4000·0–6900·0	5754·5	671·0	4670·0–7000·0	0·285
Length (cm)	57·8	3·3	51·0–62·5	58·2	2·3	54·0–62·0	0·838
Head circumference (cm)	39·9	0·9	38·0–41·5	39·4	1·7	36·5–42·0	0·327
Weight for length *z*-score at 2 months	0·25	1·01	–1·32–2·29	0·64	1·20	–1·21–3·44	0·279

* *P* < 0·05.

** *P* < 0·01.

*** *P* < 0·001.

Table 2 shows leptin, ghrelin, adiponectin and IGF-1 hormone levels in pre-feed and post-feed breast milk between groups. Breast milk ghrelin levels were the most significantly affected appetite regulating hormone during lactation. Pre-feed breast milk contained significantly higher levels of ghrelin both in mothers with obesity and normal weight (*P* = 0·000; *P* = 0·006, respectively). Although its concentration was significantly decreased during lactating in both groups, mothers with obesity had a higher level of pre-feed breast milk ghrelin than mothers with normal weight (*P* = 0·025). While the level of adiponectin in breast milk increased significantly from pre-feed breast milk to post-feed breast milk in both groups, the level of IGF-1 decreased significantly (*P* = 0·030; *P* = 0·002, respectively). Even though adiponectin levels were found to be lower in mothers with obesity, it was only significant in post-feed breast milk (*P* = 0·010). The adiponectin level in post-feed breast milk was 13·95 (sd 0·25) ng/ml in mothers with normal weight and 12·84 (sd 2·33) ng/ml mothers with obesity (*P* = 0·010). Leptin was the only appetite regulating hormone that was not affected by maternal BMI or lactation stage in our study.

**Table 2.** Leptin, ghrelin, adiponectin and IGF-1 hormones levels in breast milk of mothers with normal weight and obesity (Mean values and standard deviations)

	Normal weight (*n* 20)				Obese (*n* 20)							
	Pre-feed breast milk (Min–Max)		Post-feed breast milk (Min–Max)		Pre-feed breast milk (Min–Max)		Post-feed breast milk (Min–Max)					
Hormones	Mean	sd	Mean	sd	Mean	sd	Mean	sd	*P* ^1^	*P* ^2^	*P* ^3^	*P* ^4^
Leptin (ng/ml)	0·13	0·08	0·13	0·19	0·16	0·10	0·09	0·06	0·655	0·637	0·302	0·162
Ghrelin (pg/ml)	191·75	79·26	35·26	35·79	253·98	94·12	61·27	54·49	0·025*	0·339	0·006**	0·000***
Adiponectin (ng/ml)	11·49	1·74	13·95	0·25	10·93	2·12	12·84	2·33	0·168	0·010*	0·000***	0·000***
IGF-1 (ng/ml)	0·24	0·07	0·21	0·11	0·29	0·11	0·20	0·07	0·056	0·756	0·030*	0·002**

IGF-1, insulin-like growth factor 1; *P* ^1^, difference between mothers with normal weight and obesity in pre-feed breast milk; *P* ^2^, difference between mothers with normal weight and obesity in post-feed breast milk; *P* ^3^, difference between pre-feed and post-feed breast milk in mothers with normal weight; *P* ^4^, difference between pre-feed and post-feed breast milk in mothers with obesity.

* *P* < 0·05.

** *P* < 0·01.

*** *P* < 0·001.

When the correlation between leptin, ghrelin, adiponectin and IGF-1 hormones in breast milk and the infant’s weight for length *z*-score at 2 months was examined, no significant relationship was found (Table 3).

**Table 3.** Correlation of infant’s weight for length *z*-score at 2 months and appetite hormones in breast milk

	Infant weight for length *z*-score at 2 months			
	Normal weight (*n* 20)		Obese (*n* 20)	
	*r*	*P*	*r*	*P*
Pre-feed breast milk				
Leptin	–0·324	0·163	–0·326	0·160
Ghrelin	0·129	0·610	0·074	0·755
Adiponectin	–0·003	0·990	–0·248	0·291
IGF-1	–0·149	0·531	–0·346	0·136
Post-feed breast milk				
Leptin	–0·019	0·938	–0·420	0·065
Ghrelin	0·800	0·200	0·466	0·093
Adiponectin	0·032	0·895	–0·280	0·232
IGF-1	0·015	0·950	–0·476	0·034

Spearman correlation analysis. IGF-1, insulin-like growth factor 1.

Linear regression analyses for leptin, ghrelin, adiponectin and IGF-1 hormones in breast milk are presented in Table 4. When the factors that could affect leptin, ghrelin, adiponectin and IGF-1 hormone levels in breast milk (normal/obese, maternal age, infant sex, gestational weight gain, maternal BMI at 2 months postpartum and parity) were evaluated, the model for ghrelin in pre-feed breast milk explained 41·2 % of the variation in the dependent variable and was significant at 5 % (*r* ^2^ 0·412; *P* = 0·008). It was determined that parity is an important predictor of ghrelin in pre-feed breast milk (*P* = 0·025), that is, an increase of 1 unit in the parity caused an increase in the ghrelin level in pre-feed breast milk by 47·607 units. Furthermore, maternal age was found to be an important predictor of estimation of adiponectin, and ghrelin levels in post-feed breast milk (*P* = 0·033; *P* = 0·039, respectively).

**Table 4.** Linear regression analysis for leptin, ghrelin, adiponectin and IGF-1 hormones in breast milk

Model	*β*	*t*	*P*
Adiponectin (pre-feed breast milk)			
Normal/obese	–1·196	–1·029	0·311
Maternal age	0·153	1·891	0·067
Infant sex	0·767	1·185	0·244
Gestational weight gain	–0·046	–0·812	0·423
Maternal BMI (2 months postpartum)	0·045	0·420	0·677
Parity	–0·228	–0·455	0·652
*r* ^2^ = 0·158; *P* = 0·420			
Adiponectin (post-feed breast milk)			
Normal/obese	–1·477	–1·542	0·133
Maternal age	0·149	2·228	0·033*
Infant sex	0·932	1·748	0·090
Gestational weight gain	0·003	0·062	0·951
Maternal BMI (2 months postpartum)	0·043	0·489	0·628
Parity	–0·178	–0·430	0·670
*r* ^2^ = 0·281; *P* = 0·074			
Ghrelin (pre-feed breast milk)			
Normal/obese	–27·564	–0·583	0·564
Maternal age	2·402	0·711	0·482
Infant sex	9·556	0·359	0·722
Gestational weight gain	–1·154	–0·504	0·618
Maternal BMI (2 months postpartum)	6·187	1·460	0·154
Parity	47·607	2·359	0·025*
*r* ^2^ = 0·412; *P* = 0·008**			
Ghrelin (post-feed breast milk)			
Normal/obese	38·380	0·702	0·497
Maternal age	11·781	2·340	0·039*
Infant sex	70·696	2·142	0·055
Gestational weight gain	–0·762	–0·427	0·677
Maternal BMI (2 months postpartum)	0·148	0·047	0·964
Parity	–36·453	–1·061	0·312
*r* ^2^ = 0·438; *P* = 0·288			
IGF 1 (pre-feed breast milk)			
Normal/obese	0·025	0·451	0·655
Maternal age	0·002	0·491	0·626
Infant sex	0·003	0·092	0·927
Gestational weight gain	–0·003	–1·175	0·248
Maternal BMI (2 months postpartum)	0·004	0·802	0·428
Parity	–0·044	–1·823	0·077
*r* ^2^ = 0·181; *P* = 0·322			
IGF 1 (post-feed breast milk)			
Normal/obese	–0·026	–0·474	0·639
Maternal age	–0·002	–0·591	0·559
Infant sex	–0·047	–1·575	0·125
Gestational weight gain	–0·001	–0·197	0·845
Maternal BMI (2 months postpartum)	0·003	0·525	0·603
Parity	–0·022	–0·939	0·355
*r* ^2^ = 0·135; *P* = 0·535			
Leptin (pre-feed breast milk)			
Normal/obese	–0·019	–0·470	0·642
Maternal age	0·002	0·831	0·412
Infant sex	–0·024	–1·046	0·303
Gestational weight gain	–0·004	–2·048	0·049*
Maternal BMI (2 months postpartum)	0·004	0·984	0·332
Parity	–0·031	–1·746	0·090
*r* ^2^ = 0·196; *P* = 0·268			
Leptin (post-feed breast milk)			
Normal/obese	–0·046	–0·846	0·404
Maternal age	0·001	0·349	0·729
Infant sex	–0·042	–1·403	0·170
Gestational weight gain	–0·002	–0·912	0·368
Maternal BMI (2 months postpartum)	0·006	1·239	0·224
Parity	–0·035	–1·507	0·141
*r* ^2^ = 0·148; *P* = 0·468			

IGF-1, insulin-like growth factor 1.

* *P* < 0·05.

** *P* < 0·01.

## Discussion

Breast milk composition is widely studied since its content is crucial for an infant’s growth and development. One of the main aims of this study was to compare the pre-feed and post-feed breast milk leptin, ghrelin, adiponectin and IGF-1 hormone levels of mothers with different BMI, and we have revealed several intriguing results. We have found significant differences in ghrelin concentration in pre-feed breast milk regardless of BMI. Additionally, mothers with obesity had significantly higher ghrelin levels in pre-feed breast milk than mothers with normal weight, which could promote food intake in their infants. This might result in a greater breast milk consumption and weight gain. Although there is a general consensus over the protective effects of breast milk^(20,21)^, recent studies raise some questions about the effects of breast milk and its metabolites as a potential contributor of early childhood obesity, in particular the transmission of obesity from mother to child^(16,22–24)^. Kon *et al*. have shown that infants with high weight gain had higher ghrelin concentration in breast milk compared to infants with low and normal weight gain in the first month of lactation. Therefore, they assumed that the high ghrelin level in breast milk might be related to higher weight gain in an infant by stimulating appetite^(16)^. However, the association between maternal BMI and breast milk ghrelin concentration is inconclusive^(17,25)^.

Our results indicate that mothers with obesity had higher ghrelin levels in pre-feed breast milk and lower adiponectin levels in post-feed breast milk than mothers with normal weight. Even though adiponectin levels were significantly decreased from pre-feed breast milk to post-feed breast milk, mothers with obesity had significantly higher post-feed breast milk adiponectin concentrations. Adiponectin in breast milk has been associated with lower infant weight in the first 6 months of life^(26)^. In a previous study, a positive relationship was found between serum adiponectin level and daily weight gain of infants, while no relation was found between breast milk adiponectin concentration and daily weight gain of the infant^(27)^. According to our results, we concluded that infants of mothers with obesity were eager to consume more breast milk, which resulted in more weight gain. However, additional research is needed to further investigate this finding.

Although the functions of leptin, ghrelin and adiponectin in appetite regulation are well known, there has been no general consensus on their breast milk concentrations since previous studies found conflicting results for skimmed milk^(13,28–31)^. Additionally, the effects of maternal BMI on breast milk appetite hormone concentrations are very limited^(28,32–35)^. This contradiction could be explained by the dynamic contents of breast milk and variances in research methodology, which makes difficult to interpret and hard to compare the study results.

This research found no significant difference in breast milk leptin concentrations between mothers with obesity and normal weight. Although Andreas *et al.* found a positive association between maternal BMI and breast milk leptin concentration, leptin levels remained similar between groups^(18)^. Also, leptin levels were not changed from pre-feed and post-feed breast milk, which is consistent with the literature^(36)^. Literature suggests that breast milk leptin concentration is positively correlated with maternal adiposity and prone to increase with body weight^(22,35)^. As BMI is not a direct measure of adiposity, this might explain why leptin concentrations are not different in this study^(22,37)^.

In this study, breast milk IGF-1 levels were not significantly different in mothers with obesity and mothers with normal weight. Moreover, IGF-1 in pre-feed breast milk was found to be significantly higher in both groups. Previous work by Galante *et al.* reported that higher milk IGF-1 was associated with higher weight at 13 months^(38)^. According to a previous study, infants born to diabetic mothers were found to have greater anthropometric measurements and higher breast milk IGF-1 levels. Therefore, a positive correlation was demonstrated between breast milk IGF-1 levels and infant body weight^(39)^. However, when the correlation between infant’s weight for length *z*-score at 2 months and hormones was examined, no relationship was found. While the majority of the studies found a positive relationship between breast milk IGF-1 concentration and growth in early life, the impact of breast milk IGF-1 on endogenous IGF-1 synthesis is not known, and it is the serum concentration that promotes weight gain and adiposity.

In regression analysis, parity was found to be an important predictor of ghrelin level in pre-feed breast milk. Many maternal factors are demonstrated to influence breast milk nutrient composition, including the stage of lactation, the genetic background of the mother, parity, age and health status^(38,40–42)^. Increased breast milk ghrelin levels promote food intake^(25)^, and parity has been suggested to be a risk factor for the development of obesity^(43)^. The positive relationship we demonstrated between parity and pre-feed breast milk ghrelin levels in the regression analysis supports these previous findings. The increase in pre-feed breast milk ghrelin level with parity can also explain the increase in food consumption and obesity in infants. However, this finding needs to be explored further.

There were also some limitations in the study. Firstly, we could not support our results with blood hormone tests of neither mothers nor infants since drawing blood from infants needs several ethical consents. Having data on at least the maternal blood hormone tests, which include insulin, would have strengthened the results. Secondly, we were not able to measure the actual adiposity of mothers, which could have expanded our results and brought a new perspective into the study. Thirdly, we could not follow up the infants to establish the associations between the concentrations of these hormones and the subsequent likelihood of infant overweight/obesity. Also, we could not systematically question maternal diet, which could affect breast milk composition. Finally, measures of milk components in skim milk warrant further caution in this case as the literature shows there are more components in whole breast milk than in skim milk, and it is the whole milk that infants receive.

## Conclusion

In conclusion, this study indicates that pre-pregnancy BMI is associated with leptin, ghrelin, adiponectin and IGF-1 levels. According to the results of this study, maternal BMI might be associated with the appetite regulating hormone levels in breast milk. Hence, further longitudinal research is needed to better understand the effect of leptin, ghrelin, adiponectin and IGF-1 on infants’ adulthood.

## References

[1] World Health Organization (2020) Children: New Threats to Health. https://www.who.int/news-room/fact-sheets/detail/children-new-threats-to-health (accessed January 2021).

[2] World Health Organization (2020) The Global Health Observatory. https://www.who.int/data/gho/data/themes/topics/joint-child-malnutrition-estimates-unicef-who-wb (accessed May 2021).

[3] Catalano PM & Kirwan JP (2001) Maternal factors that determine neonatal size and body fat. Curr Diab Rep 1, 71–77. 12762960 10.1007/s11892-001-0013-y

[4] Carlsen EM , Renault KM , Nørgaard K , et al. (2014) Newborn regional body composition is influenced by maternal obesity, gestational weight gain and the birthweight standard score. Acta Paediatr 103, 939–945. 24942370 10.1111/apa.12713

[5] Leddy MA , Power ML & Schulkin J (2008) The impact of maternal obesity on maternal and fetal health. Rev Obstet Gynecol 1, 170–178. 19173021 PMC2621047

[6] De Luca A , Frasquet-Darrieux M , Gaud MA , et al. (2016) Higher leptin but not human milk macronutrient concentration distinguishes normal-weight from obese mothers at 1-month postpartum. PLoS One 11, e0168568. 28005966 10.1371/journal.pone.0168568 PMC5179069

[7] Ruager-Martin R , Hyde MJ & Modi N (2010) Maternal obesity and infant outcomes. Early Hum Dev 86, 715–722. 20846795 10.1016/j.earlhumdev.2010.08.007

[8] Wilson RM & Messaoudi I (2015) The impact of maternal obesity during pregnancy on offspring immunity. Mol Cell Endocrinol 418, 134–142. 26232506 10.1016/j.mce.2015.07.028 PMC4674375

[9] Panagos PG , Vishwanathan R , Penfield-Cyr A , et al. (2016) Breastmilk from obese mothers has pro-inflammatory properties and decreased neuroprotective factors. J Perinatol 36, 284–290. 26741571 10.1038/jp.2015.199 PMC4888773

[10] Gila-Diaz A , Arribas SM , Algara A , et al. (2019) A review of bioactive factors in human breastmilk: a focus on prematurity. Nutrients 11, 1307. 10.3390/nu11061307 PMC6628333 31185620

[11] Zheng M , Lamb KE , Grimes C , et al. (2018) Rapid weight gain during infancy and subsequent adiposity: a systematic review and meta-analysis of evidence. Obes Rev 19, 321–332. 29052309 10.1111/obr.12632 PMC6203317

[12] Kugananthan S , Gridneva Z , Lai CT , et al. (2017) Associations between maternal body composition and appetite hormones and macronutrients in human milk. Nutrients 9, 252. 10.3390/nu9030252 PMC5372915 28282925

[13] Eilers E , Ziska T , Harder T , et al. (2011) Leptin determination in colostrum and early human milk from mothers of preterm and term infants. Early Hum Dev 87, 415–419. 21482454 10.1016/j.earlhumdev.2011.03.004

[14] Gridneva Z , Kugananthan S , Rea A , et al. (2018) Human milk adiponectin and leptin and infant body composition over the first 12 months of lactation. Nutrients 10, 1125. 10.3390/nu10081125 PMC6115716 30127292

[15] Karatas Z , Durmus Aydogdu S , Dinleyici EC , et al. (2011) Breastmilk ghrelin, leptin, and fat levels changing foremilk to hindmilk: is that important for self-control of feeding? Eur J Pediatr 170, 1273–1280. 21384109 10.1007/s00431-011-1438-1

[16] Kon IY , Shilina NM , Gmoshinskaya MV , et al. (2014) The study of breast milk IGF-1, leptin, ghrelin and adiponectin levels as possible reasons of high weight gain in breast-fed infants. Ann Nutr Metab 65, 317–323. 25402263 10.1159/000367998

[17] Badillo-Suárez PA , Rodríguez-Cruz M & Nieves-Morales X (2017) Impact of metabolic hormones secreted in human breast milk on nutritional programming in childhood obesity. J Mammary Gland Biol Neoplasia 22, 171–191. 28653126 10.1007/s10911-017-9382-y

[18] Andreas NJ , Hyde MJ , Gale C , et al. (2014) Effect of maternal body mass index on hormones in breast milk: a systematic review. PLoS One 9, e115043. 25536196 10.1371/journal.pone.0115043 PMC4275218

[19] World Health Organization (2021) Body Mass Index – BMI. https://www.euro.who.int/en/health-topics/disease-prevention/nutrition/a-healthy-lifestyle/body-mass-index-bmi (accessed January 2021).

[20] Horta BL , Loret de Mola C & Victora CG (2015) Long-term consequences of breastfeeding on cholesterol, obesity, systolic blood pressure and type 2 diabetes: a systematic review and meta-analysis. Acta Paediatr 104, 30–37. 26192560 10.1111/apa.13133

[21] Hess C , Ofei A & Mincher A (2015) Breastfeeding and childhood obesity among African Americans: a systematic review. MCN Am J Matern Child Nurs 40, 313–319. 26295508 10.1097/NMC.0000000000000170

[22] Schneider-Worthington CR , Bahorski JS , Fields DA , et al. (2020) Associations among maternal adiposity, insulin, and adipokines in circulation and human milk. J Hum Lact 890334420962711. 10.1177/0890334420962711 PMC8276526 33035124

[23] Isganaitis E , Venditti S , Matthews TJ , et al. (2019) Maternal obesity and the human milk metabolome: associations with infant body composition and postnatal weight gain. Am J Clin Nutr 110, 111–120. 30968129 10.1093/ajcn/nqy334 PMC6599743

[24] Lagström H , Rautava S , Ollila H , et al. (2020) Associations between human milk oligosaccharides and growth in infancy and early childhood. Am J Clin Nutr 111, 769–778. 32068776 10.1093/ajcn/nqaa010 PMC7138667

[25] Fields DA , Schneider CR & Pavela G (2016) A narrative review of the associations between six bioactive components in breast milk and infant adiposity. Obesity 24, 1213–1221. 27151491 10.1002/oby.21519 PMC5325144

[26] Newburg DS , Woo JG & Morrow AL (2010) Characteristics and potential functions of human milk adiponectin. J Pediatr 156, S41–S46. 20105665 10.1016/j.jpeds.2009.11.020 PMC2875873

[27] Cesur G , Ozguner F , Yilmaz N , et al. (2012) The relationship between ghrelin and adiponectin levels in breast milk and infant serum and growth of infants during early postnatal life. J Physiol Sci: JPS 62, 185–190. 22311236 10.1007/s12576-012-0193-z PMC10717336

[28] Andreas NJ , Hyde MJ , Herbert BR , et al. (2016) Impact of maternal BMI and sampling strategy on the concentration of leptin, insulin, ghrelin and resistin in breast milk across a single feed: a longitudinal cohort study. BMJ Open 6, e010778. 10.1136/bmjopen-2015-010778 PMC4947729 27388351

[29] Schueler J , Alexander B , Hart AM , et al. (2013) Presence and dynamics of leptin, GLP-1, and PYY in human breast milk at early postpartum. Obesity 21, 1451–1458. 23408760 10.1002/oby.20345 PMC3742570

[30] Bronsky J , Mitrova K , Karpisek M , et al. (2011) Adiponectin, AFABP, and leptin in human breast milk during 12 months of lactation. J Pediatr Gastroenterol Nutr 52, 474–477. 21407103 10.1097/MPG.0b013e3182062fcc

[31] Savino F , Sorrenti M , Benetti S , et al. (2012) Resistin and leptin in breast milk and infants in early life. Early Hum Dev 88, 779–782. 22641277 10.1016/j.earlhumdev.2012.05.004

[32] Sims CR , Lipsmeyer ME , Turner DE , et al. (2020) Human milk composition differs by maternal BMI in the first 9 months postpartum. Am J Clin Nutr 112, 548–557. 32401302 10.1093/ajcn/nqaa098 PMC7458771

[33] Chan D , Goruk S , Becker AB , et al. (2018) Adiponectin, leptin and insulin in breast milk: associations with maternal characteristics and infant body composition in the first year of life. Int J Obes 42, 36–43. 10.1038/ijo.2017.189 28925410

[34] Enstad S , Cheema S , Thomas R , et al. (2021) The impact of maternal obesity and breast milk inflammation on developmental programming of infant growth. Eur J Clin Nutr 75, 180–188. 32814855 10.1038/s41430-020-00720-5 PMC7855210

[35] Sadr Dadres G , Whitaker KM , Haapala JL , et al. (2019) Relationship of maternal weight status before, during, and after pregnancy with breast milk hormone concentrations. Obesity 27, 621–628. 30900412 10.1002/oby.22409 PMC6432940

[36] Larson-Meyer DE , Schueler J , Kyle E , et al. (2020) Appetite-regulating hormones in human milk: a plausible biological factor for obesity risk reduction? J Hum Lact 890334420954160. 10.1177/0890334420954160 33030994

[37] Nuttall FQ (2015) Body Mass Index: obesity, BMI, and health: a critical review. Nutr Today 50, 117–128. 27340299 10.1097/NT.0000000000000092 PMC4890841

[38] Galante L , Pundir S , Lagström H , et al. (2020) Growth factor concentrations in human milk are associated with infant weight and BMI from birth to 5 years. Front Nutr 7, 110. 32850934 10.3389/fnut.2020.00110 PMC7403458

[39] Mohsen AH , Sallam S , Ramzy MM , et al. (2016) Investigating the relationship between Insulin-like Growth Factor-1 (IGF-1) in diabetic mother’s breast milk and the blood serum of their babies. Electron Phys 8, 2546–2550. 10.19082/2546 PMC4965206 27504171

[40] Kocaadam B , Koksal E , Ozcan KE , et al. (2019) Do the adiponectin and leptin levels in preterm and term breast milk samples relate to infants’ short-term growth? J Dev Orig Health Dis 10, 253–258. 30203736 10.1017/S2040174418000703

[41] Martin LJ , Woo JG , Geraghty SR , et al. (2006) Adiponectin is present in human milk and is associated with maternal factors. Am J Clin Nutr 83, 1106–1111. 16685053 10.1093/ajcn/83.5.1106

[42] Ng PC , Lee CH , Lam CW , et al. (2005) Ghrelin in preterm and term newborns: relation to anthropometry, leptin and insulin. Clin Endocrinol 63, 217–222. 10.1111/j.1365-2265.2005.02328.x 16060917

[43] Jenum AK , Sommer C , Sletner L , et al. (2013) Adiposity and hyperglycaemia in pregnancy and related health outcomes in European ethnic minorities of Asian and African origin: a review. Food Nutr Res 57, 18889. 10.3402/fnr.v57i0.18889 PMC3585772 23467680

